# Preventing Human Papillomavirus and Vaccine Strategies

**DOI:** 10.3390/vaccines14030270

**Published:** 2026-03-18

**Authors:** Rui M. Gil da Costa

**Affiliations:** 1 Department of Pathology, State University of Maranhão, São Luís 65055-310, Brazil; ruioliveira@cca.uema.br; 2 Research Center for Experimental and Clinical Physiopathology and Pharmacology (NEC), Federal University of Maranhão (UFMA), São Luís 65080-805, Brazil; 3 Centre for the Research and Technology of Agroenvironmental and Biological Sciences, CITAB, Inov4Agro, Universidadede Trás-os-Montes e Alto Douro, UTAD, Quinta de Prados, 5000-801 Vila Real, Portugal; 4 Molecular Oncology and Viral Pathology Group, Research Center of IPO Porto (CI-IPOP)/RISE@CI-IPOP (Health Research Network), Portuguese Oncology Institute of Porto (IPO Porto), Porto Comprehensive Cancer Center (Porto.CCC), 4200-072 Porto, Portugal; 5 LEPABE—Laboratory for Process Engineering, Environment, Biotechnology and Energy, AliCE—Associate Laboratory in Chemical Engineering, Faculty of Engineering, University of Porto, Rua Dr. Roberto Frias, 4200-465 Porto, Portugal

## 1. Introduction

Vaccination against human papillomavirus (HPV) has been widely adopted, aiming at preventing infection and, ultimately, the development of HPV-driven uterine cervical neoplasia [1]. HPV vaccines are safe and highly effective in preventing cervical cancer, fueling hope that this malignancy could be eradicated in the future [2]. However, vaccination efforts have had different levels of success in different regions around the world, and barriers to effective implementation, including lack of public awareness and infrastructure, remain in many countries [3,4]. Additionally, HPV also causes a significant proportion of oropharyngeal [5] and ano-genital squamous cell carcinoma cases, including penile, anal, vulvar, and vaginal cancers [6]. The data concerning the efficacy of HPV vaccination programs on preventing these types of cancer are limited, largely due to their comparatively lower incidence [2].

Long before the implementation of HPV vaccines, cervical cancer screening programs were already essential for preventing this disease by detecting early lesions in cytological smears. These programs continue to be essential even after HPV vaccines have been extensively adopted. However, screening methods now include PCR-based techniques for detecting and genotyping HPV, either as an alternative to or in combination with the analysis of cytological smears [7,8]. The effective implementation, evaluation, and equitable access to these technologies across social and geographical barriers remain important issues in the field of HPV prevention.

This Special Issue aimed to address the major issues related to HPV prevention and vaccination and includes five accepted manuscripts, including three research articles and two systematic reviews.

## 2. An Overview of Published Articles

The three research articles addressed different questions, ranging from risk factors for HPV infection, to the efficacy of HPV vaccines in inducing effective antibody titers to a phase 1 clinical trial of a new HPV vaccine.

Wiek et al. (contribution 1) investigated vaccine-induced anti-HPV18 immunoglobulin (IgG) antibody titers and subsequent HPV18/45 infections in a nested matched case–control study focused on a prospective longitudinal cohort of adolescent and young adult women vaccinated with the quadrivalent HPV vaccine. The findings from the study suggest that higher IgG antibody levels to HPV16/18 after vaccination represent an increased likelihood of protection from homologous and cross-reactive HPV types (HPV16/18/31/45). Additionally, the authors suggest that differences in antibody titers are associated with breakthrough infection after vaccination and propose further studies on long-term antibody titers and their implications for breakthrough infections.

Wei et al. (contribution 2) conducted a dose-escalating phase 1 clinical study to evaluate the safety, tolerability, and immunogenicity of an *Escherichia coli*-expressed recombinant nonavalent HPV vaccine in women aged 18–145 years old. The authors found that no serious adverse effects occurred, but the low-dose group receiving the new recombinant vaccine experienced significantly more adverse reactions than the control group receiving Gardasil 9. Overall, the authors concluded that the new recombinant nonavalent HPV vaccine exhibits good tolerability and strong immunogenicity and propose further efficacy studies in larger populations.

Jensen et al. (contribution 3) employed a survey asking demographic, general and sexual health, and HPV-related questions, which was emailed to 28,095 students at a United States of America University, to study HPV vaccination patterns, uptake, perceptions, and sexual risk factors. The survey had a response rate of 4.9% with a large majority of respondents being female. The authors report that vaccine uptake was associated with being female, studying health sciences, being sexually active, and having been vaccinated against COVID-19 or influenza. Conversely, parental preference was the main reason for being unvaccinated. The authors conclude that education regarding HPV vaccination must be improved, particularly in males, those who are not sexually active, and those who did not receive other vaccines.

The two systematic review articles addressed issues related to the uptake of HPV vaccines, including challenges that preclude wider vaccine uptake and possible strategies to overcome those challenges.

Tobaiqy and MacLure (systematic 4) conducted a systematic review aiming to identify the primary challenges associated with HPV vaccination, propose effective strategies to improve vaccination uptake, and compile relevant evidence into a comprehensive overview to inform policy and practice. Based on the scientific literature published between 2000 and 2024, the authors list five strategies to improve vaccine uptake, namely, improving parental and school engagement, using technology and multimedia tools, promoting the healthcare providers’ role, developing multicomponent interventions, and providing targeted interventions for immigrant groups. Additionally, the authors emphasize the need for a multifaceted approach to increase vaccination rates.

Chandeying and Thongseiratch (contribution 5) explored the potential of interventions based on patient’s electronic medical records on the uptake of HPV vaccines. The results from the study suggest that such interventions increase the initiation and completion of the HPV vaccination schedule. Additionally, the authors state that improving provider feedback and parental education increase vaccine uptake and suggest that the broader adoption of digital health technologies in vaccination programs will contribute to their success.

## 3. Conclusions

Overall, the research articles and systematic reviews included in this Special Issue addressed key issues related to the prevention of HPV infection and the successful implementation of HPV vaccines. Increasing vaccine uptake and the completion of vaccination schedules remain significant challenges that may be addressed in various ways. Much work is needed concerning these issues, and research groups across the globe will continue to use their diverse approaches achieve more balanced health gains. Ultimately, implementing improved vaccination programs with effective screening will lead to the eradication of cervical cancer.

## Data Availability

No new data were generated in this study.

## Abbreviations

The following abbreviations are used in this manuscript:

COVID-19	Coronavirus Disease of 2019
HPV	Human Papillomavirus
IgG	Immunoglobulin G
